# Spatial variability of prokaryotic and viral abundances in the Kermadec and Atacama Trench regions

**DOI:** 10.1002/lno.11711

**Published:** 2021-02-28

**Authors:** Clemens Schauberger, Mathias Middelboe, Morten Larsen, Logan M. Peoples, Douglas H. Bartlett, Finn Kirpekar, Ashley A. Rowden, Frank Wenzhöfer, Bo Thamdrup, Ronnie N. Glud

**Affiliations:** ^1^ Department of Biology, Nordcee and HADAL University of Southern Denmark Odense Denmark; ^2^ Marine Biological Section, Department of Biology University of Copenhagen Helsingør Denmark; ^3^ Marine Biology Research Division, Scripps Institution of Oceanography University of California San Diego La Jolla California USA; ^4^ Department of Biochemistry and Molecular Biology University of Southern Denmark Odense M Denmark; ^5^ National Institute of Water and Atmospheric Research Wellington New Zealand; ^6^ School of Biological Sciences, Victoria University of Wellington Wellington New Zealand; ^7^ Alfred Wegener Institute, Helmholtz Center for Polar and Marine Research Bremerhaven Germany; ^8^ Max Planck Institute for Marine Microbiology and Ecology Bremen Germany; ^9^ Department of Ocean and Environmental Sciences Tokyo University of Marine Science and Technology Tokyo Japan; ^10^ Danish Institute for Advanced Study – DIAS, University of Southern Denmark Odense Denmark

## Abstract

Hadal trenches represent the deepest part of the ocean and are dynamic depocenters with intensified prokaryotic activity. Here, we explored the distribution and drivers of prokaryotic and viral abundance from the ocean surface and 40 cm into sediments in two hadal trench regions with contrasting surface productivity. In the water column, prokaryotic and viral abundance decreased with water depth before reaching a rather stable level at ~ 4000 m depth at both trench systems, while virus to prokaryote ratios were increasing with depth, presumably reflecting the declining availability of organic material. Prokaryotic and viral abundances in sediments were lower at the adjacent abyssal sites than at the hadal sites and declined exponentially with sediment depth, closely tracking the attenuation of total organic carbon (TOC) content. In contrast, hadal sediment exhibited erratic depth profiles of prokaryotes and viruses with many subsurface peaks. The prokaryotic abundance correlated well to extensive fluctuations in TOC content at centimeter scale, which were likely caused by recurring mass wasting events. Yet while prokaryotic and viral abundances cross correlated well in the abyssal sediments, there was no clear correlation in the hadal sites. The results suggested that dynamic depositional conditions and higher substrate availability result in a high spatial heterogeneity in viral and prokaryotic abundances in hadal sediments in comparison to more stable abyssal settings. We argue that these conditions enhance the relatively importance of viruses for prokaryotic mortality and carbon recycling in hadal settings.

Pelagic carbon fluxes generally decrease with increasing oceanic depth and can be approximated by simple power functions known as Martin curves (Martin et al. 1987). The reduction in organic carbon availability with increasing water depth leads to a decrease in pelagic prokaryotic abundances (Azam 1998; Parada et al. 2007) from up to 1 × 10^7^ prokaryotes mL^−1^ in eutrophic surface waters to less than 1 × 10^4^ prokaryotes mL^−1^ at abyssal water depths (e.g., Wigington et al. 2016). Sediments, where organic carbon accumulates, host prokaryotic abundances that are 2–3 orders of magnitude higher than the pelagic levels, with typical values of 1 × 10^9^ to 5 × 10^9^ prokaryotes mL^−1^ in coastal surface sediments compared to 5 × 10^6^ prokaryotes mL^−1^ in open ocean abyssal sediments (e.g., Kallmeyer et al. 2012). However, recent studies suggest that the benthic decline in prokaryotic abundance might be reversed in some hadal settings (Danovaro et al. 2003; Glud et al. 2013; Manea et al. 2019). For instance, sediments in the central basins of the Mariana and Tonga trenches act as prokaryotic hotspots with elevated abundances and intensified diagenetic activities as compared to nearby abyssal settings (Glud et al. 2013; Wenzhöfer et al. 2016). This apparent increase at hadal depths is presumed to be related to intensified deposition dynamics in trench systems, facilitated by mass wasting, downslope funneling, and fluid dynamics that enrich labile organic material in the central basins (Itou et al. 2000; Turnewitsch et al. 2014; Ichino et al. 2015). However, there is increasing evidence that hadal seascapes and local deposition dynamics result in highly variable conditions along the trench axes and that central basins may not represent hadal environments in general (Stewart and Jamieson 2018).

Due to the inherent technical challenge of sampling in the hadal zone, there are only few reports on prokaryotic abundance or activity from great depths (Liu et al. 2018). Deep‐ocean prokaryotes appear adapted to their in situ conditions (Tamburini et al. 2013) and it has been shown that decompression and transient heating during sample recovery from great oceanic depth may induce artifacts that affect solute concentrations and prokaryotic activities (Chastain and Yayanos 1991; Glud et al. 1994; Hall et al. 2007), but it remains unknown if sample recovery from hadal depths also affects the quantification of prokaryotes and virus abundances.

Viral abundance generally exceeds prokaryotic abundance, and large variations in the virus to prokaryote (VP) ratio (from 3 to 160 in pelagic marine systems), suggest large differences in the role of viral infections across marine ecosystems (Wigington et al. 2016). Viruses act as an important mortality factor for prokaryotes, function as a shortcut for recycling organic carbon in the microbial loop (Suttle 2007), and are drivers of prokaryotic diversification (Martiny et al. 2014). VP ratios generally increase with decreasing cell density (Wigington et al. 2016) and are elevated in deep waters (Parada et al. 2007). These patterns, in combination with the decrease in the relative contribution of eukaryotic grazing to prokaryotic mortality with oceanic depth (Rocke et al. 2015), hints an increase in importance of viruses below epipelagic depths (> 1000 m). The situation is similar for bathyal and abyssal sediments, where an estimated virus‐induced lysis of up to 80% of heterotrophic prokaryotic production (Danovaro et al. 2008) has emphasized the potential contribution of viral activity for prokaryotic mortality and benthic diagenetic activity (Middelboe et al. 2006). However, data at oceanic depths exceeding 6000 m are scarce (Wigington et al. 2016) and the abundance and importance of viruses in pelagic and benthic hadal settings remain largely unknown (Danovaro et al. 2016; Manea et al. 2019; Rastelli et al. 2019).

As part of the broader coordinated effort to explore the biogeochemistry and ecology of hadal trenches, we here provide the first systematic investigation of prokaryotic and viral abundances in hadal waters and sediments, including samples from the ocean surface to 40 cm sediment depth at multiple sites in two contrasting hadal environments; the Kermadec and Atacama Trenches. We evaluate potential sample recovery artifacts and use our data to discuss key drivers of prokaryotic and viral distribution, and assess the overall importance of viruses in the hadal realm.

## Material and methods

### Study area and sampling sites

**Fig. 1:**
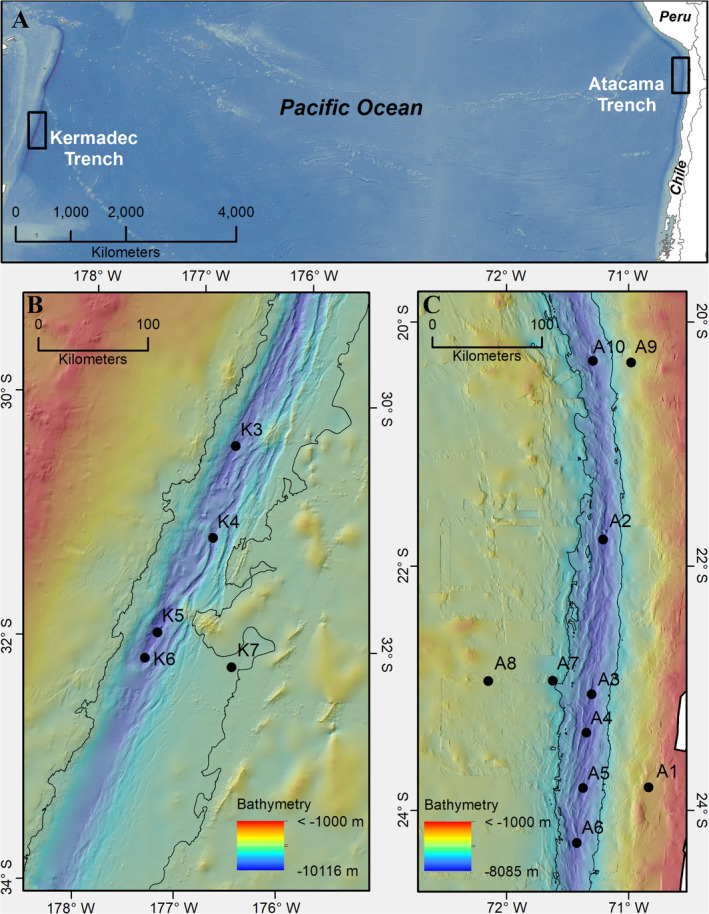
Maps of the regions studied (black boxes) in the Kermadec Trench and Atacama Trench (**A**), bathymetric maps with sampling sites (black circles) in the Kermadec Trench (**B**), and Atacama Trench (**C**). All bathymetry data were sourced from the Global Multi‐Resolution Topography Synthesis (Ryan et al. 2009).

We targeted two geographically distant hadal trenches underlying water columns with contrasting productivity levels (Fig. 1A). The Kermadec Trench (Fig. 1B) has a maximum depth of 10,047 m and is located in a relatively oligotrophic region of the southwest Pacific with primary productivity of around 380 mg C m^−2^ d^−1^ (Lutz et al. 2007). Here we sampled four hadal sites (K3–K6) on a ~ 210 km long transect along the axis of the ~ 1500 km long and ~ 60 km wide Kermadec Trench (Angel 1982) and one off‐axis site (K7) on the border of the abyssal realm at the subducting plate. The Atacama Trench off Chile (Fig. 1C) includes the deepest location in the southeastern Pacific Ocean with a maximum depth of 8055 m and underlies the Humboldt Current upwelling region with high average primary production of around 910 mg C m^−2^ d^−1^ (Lutz et al. 2007). Here we sampled six hadal sites (A2–A6; A10) on a ~ 430 km long transect along the southern part of the ~ 5900 km long and ~ 100 km wide Atacama Trench axis (Angel 1982), one bathyal site and one abyssal site on the continental shelf (A1 and A9, respectively) and two sites at abyssal depths on the subducting plates (A7 and A8). An overview of all sampling locations and associated oceanic depths can be found in Table 1.

**Table 1:** Sampling positions and water depths in the Kermadec and Atacama Trenches.

TAN1711	Kermadec Trench		
Site	Latitude	Longitude	Water depth (m)
K3	30°22.84′S	176°38.48′W	9540
K4	31°08.41′S	176°48.48′W	9300
K5	31°56.14′S	177°17.48′W	10,010
K6	32°08.93′S	177°23.91′W	9555
K7	32°11.22′S	176°33.66′W	6080

SO261	Atacama Trench		
Site	Latitude	Longitude	Water depth (m)
A1	23°48.72′S	70°50.04′W	2560
A2	21°46.86′S	71°12.48′W	7995
A3	23°02.94′S	71°18.12′W	7915
A4	23°21.78′S	71°20.60′W	8085
A5	23°49.02′S	71°22.32′W	7770
A6	24°15.96′S	71°25.38′W	7720
A7	22°56.22′S	71°37.08′W	5500
A8	22°56.40′S	72°08.76′W	4000
A9	20°19.97′S	70°58.70′W	4050
A10	20°19.14′S	71°17.46′W	7770

### Water column sampling

Water samples were collected with 7.5‐liter Niskin bottles for the quantification of prokaryotes and viruses. For depths down to ~ 6000 m, bottles were mounted on a deck‐controlled CTD rosette. For the hadal depth range, we applied a wired, custom‐built, hadal‐rated sampler with automatic triggering of bottle closure at preprogrammed pressure levels. Immediately after retrieval of the water samples, subsamples of 2 mL were fixed with 25% electron microscopy graded glutaraldehyde (final concentration; 1%) and stored at −80°C until quantification by flow cytometry.

### In situ fixation of water column samples

To quantify potential artifacts associated with decompression and transient heating during sample recovery from hadal depths, we applied a small, spring‐loaded syringe sampler holding two racks of four 50‐mL plastic syringes (*see* Glud et al. 1995). Two syringes were prefilled with either 5 mL glutaraldehyde (Kermadec Trench) or 6 mL of formaldehyde (Atacama Trench) and two control syringes, which were set to 0 mL and had the residual airspace filled with sterile filtered seawater. The locked spring system was released at a preset hydrostatic pressure and filled the syringes with 45 mL (Kermadec Trench) or 44 mL (Atacama Trench) hadal water. After recovery, three replicate subsamples were collected from each syringe and frozen at −80°C for later enumeration by flow cytometry (see details below).

Further tests of pressure effects on prokaryotic abundances were performed at a few selected hadal depths in the Atacama Trench using a pressure‐retaining sampler for in situ fixation (Peoples et al. 2019*a*). In short, the pressure‐retaining sampler was loaded with ~ 13 mL of formaldehyde prior to deployment and then opened at a preprogrammed pressure allowing seawater to mix with the fixative to a final concentration of ~ 3% formaldehyde before the sampler closed. Reference samples for comparison were collected in parallel using Niskin bottles and were fixed onboard at final concentrations of 3% formaldehyde. In these experiments, the prokaryotic abundances of controls and in situ fixed samples were quantified by epifluorescence microscopy.

### Sediment recovery

In the Atacama Trench and at site K4 and K6 in the Kermadec Trench, sediment cores were collected by a multicorer or an autonomous lander system. In all cases, the retrieved sediment cores had clear overlying bottom water and appeared virtually undisturbed. At Kermadec Trench sites K3, K4, K5, and K7, sediment was recovered by subsampling a boxcorer (50 × 50 cm). At sites K3, K4, and K5, the overlying water appeared turbid and pore‐water analyses of nitrate and nitrite concentrations indicated disturbance or loss of the upper ≤ 1 cm, ≤ 1 cm, and 5–7 cm, respectively, while the material sampled at site K7 had a clear overlying water phase and showed no sign of disturbance (unpublished results).

### Sediment core slicing

Upon arrival on deck, sediment cores were placed in a cold room at 3°C and quickly processed. Cores from the Kermadec Trench were sectioned in 1 cm intervals for the upper 2 cm, followed by 2 cm intervals from 2 to 10 cm and 5 cm intervals thereafter. In the Atacama Trench, the sediment exhibited steeper biogeochemical gradients (Glud et al. 2021) and thus sediment was sectioned at finer resolution; 1 cm slices down to 10 cm, 2.5 cm slices to 20 cm, followed by 5 cm slices until the bottom of the cores.

### Extraction of prokaryotes and viruses from sediment

Viruses and prokaryotes were extracted from sediments at 3°C using established protocols developed for marine sediments (Danovaro and Middelboe 2010; Corinaldesi et al. 2017). In short, 8 mL of homogenized sediment was transferred to 50 mL polypropylene centrifuge tubes and 4 mL 0.02 *μ*m filtered bottom water and 1 mL 50 mmol L^−1^ tetrasodium pyrophosphate were added. These slurries were then homogenized followed by addition of another 30 mL of 0.02 *μ*m filtered bottom water. Subsequently, the samples were sonicated at 40 kHz for 3 × 1 min with 30 s manual shaking between sonications. The tubes were then centrifuged (800 × *g*, 10 min) and supernatants recovered in 250 mL polycarbonate bottles (Nalgene). The sediment pellets were resuspended in 30 mL 0.02 *μ*m filtered bottom water, sonicated, centrifuged, and the supernatant recovered. This washing procedure was repeated, and the supernatants were combined. Duplicate subsamples of 2 mL sediment extract for prokaryote counts were transferred to cryovials, DNase I (10 U mL^−1^) was added, and vials were incubated for 15 min in darkness. The samples were then fixed with 50 *μ*L 25% glutaraldehyde and stored at −80°C for subsequent flow cytometry analysis. For viral counts, duplicate 2 mL extracts were filtered directly into cryotubes through a 0.2 *μ*m syringe filter for removal of prokaryotes and sediment particles. DNase I (10 U mL^−1^) was then added to the filtered sample and vials were incubated for 15 min in darkness. After incubation, 50 *μ*L 25% glutaraldehyde was added for fixation and samples were stored at −80°C until quantification via flow cytometry.

### Flow cytometry and assessment of in situ fixation precision

Prokaryotic and viral abundances were quantified using flow cytometry (BD FACSCanto™ II) after staining samples with SYBR Green I. Water column samples were measured in duplicates (Kermadec Trench) or triplicates (Atacama Trench) directly (Brussaard 2004), whereas duplicate sediment extracts were diluted 1 : 10 in 0.02 *μ*m‐filtered TE Buffer prior to all measurements. All samples were run using a flow rate of 5–7 *μ*L min^−1^, as determined by BD Trucount™ Beads. The flow cytometry data were subsequently analyzed using the “Flowing software suite” (flowingsoftware.btk.fi). The laser settings and gating examples can be found in Supporting Information Fig. S1 and Table S1.

The summed prokaryotic and viral abundances of controls and in situ fixed samples of all deployments were tested for significant differences by paired *t*‐tests. The standard deviations (SDs) of technical replicates of prokaryotic and viral abundance in each syringe at the Kermadec Trench and the Atacama Trench were on average 1.8 × 10^4^ prokaryotes mL^−1^ and 1.3 × 10^5^ viruses mL^−1^, and 7.4 × 10^3^ prokaryotes mL^−1^ and 1.3 × 10^5^ viruses mL^−1^, respectively. The SDs of the average differences in prokaryotic counts of in situ syringes and onboard fixed controls were 2.6 × 10^4^ prokaryotes mL^−1^ for the Kermadec Trench and 2.8 × 10^4^ prokaryotes mL^−1^ for the Atacama Trench. Given the number of replicates (Kermadec Trench: 7, Atacama Trench: 5), this allowed us to observe differences in prokaryotic abundance upon recovery as low as 2.4 × 10^4^ prokaryotes mL^−1^ in the Kermadec Trench and 2.6 × 10^4^ prokaryotes mL^−1^ in the Atacama Trench (paired *t*‐test, *p* < 0.05). In the case of viruses, we would have been able to observe differences as low as 2 × 10^5^ viruses mL^−1^ in the Kermadec Trench and 1.7 × 10^5^ viruses mL^−1^ in the Atacama Trench waters (paired *t*‐test, *p* < 0.05).

### Epifluorescence microscopy

The current study represents the first quantification of viruses from marine sediments using flow cytometry, and in order to assess the specific quantification procedure, these counts were compared with numbers obtained using classical epifluorescence microscopy (Siem‐Jørgensen et al. 2008; Suttle and Fuhrman 2010). Briefly, 500 *μ*L of viral extracts was filtered onto 25 mm, 0.02 *μ*m Anodisc filters (Fisher Scientific) that were backed with a 25 mm GF/C filter (Millipore). The filters were then stained with an 80 *μ*L drop of SYBR Green I, incubated for 20 min in darkness, washed with 0.02 *μ*m filtered Milli‐Q water and finally mounted on a slide in an antifading solution (Phosphate‐bufffered saline : glycerol mixture with 0.1% phenylenediamine). From each filter, more than 400 viruses were counted within 17–40 randomly selected fields.

### Virus morphology by transmission electron microscopy

Viral extracts and morphologies were inspected in two selected samples of surface sediment from each trench (K6, K7, A6, A7) using transmission electron microscopy. Two milliliter viral extract was centrifuged (100,000 × *g*, 90 min, 4°C; 70.1Ti; Beckman) onto formvar‐carbon‐coated copper grids and then stained with 10 *μ*L 2% sodium phosphotungstate (pH 7.4) for 2 min. Excess stain was removed by touching the edge of the grids with filter paper, and grids were washed with several drops of distilled water and allowed to dry on filter paper for 15 min. The grids were observed with a JEM‐2100 transmission electron microscope (JEOL) operated at 80 kV.

### Quantification of total organic carbon

Subsamples of the each sediment slice (see above) were used for quantification of the weight percent of total organic carbon (TOC%). The sediment was predried at 105°C for 24 h before acidification with 1 mol L^−1^ HCl. Subsequently 30–40 mg of dried sediment was packed in tin capsules that were combusted and analyzed on an elemental analyzer (Flash 2000, Thermo Fisher Scientific).

## Results

### In situ fixation experiments

The potential effect of decompression and transient heating on prokaryotic and viral counts was investigated by comparing counts in hadal water samples fixed either in situ or onboard. We performed a total of 12 paired comparisons with the spring‐loaded syringe system, recovered from 5950 to 8720 m; seven in the Kermadec Trench and five in the Atacama Trench (Supporting Information Table S2). Because the fixation procedures and overall abundances in the two trenches differed (*see* “Material and methods” section), the two data sets were assessed separately. For both data sets, there was no significant difference between prokaryotic counts of samples fixed onboard and those fixed in situ (*p* > 0.05; paired two‐tailed Welch's *t*‐test). The average differences between onboard and in situ fixation were 1.1 × 10^4^ ± 9.9 × 10^3^ prokaryotes mL^−1^ and 2.4 × 10^4^ ± 1.2 × 10^4^ prokaryotes mL^−1^ in the Kermadec Trench and the Atacama Trench, respectively (Supporting Information Table S2), corresponding to 23% and 19% lower average abundances for samples fixed in situ vs. onboard. Similarly, the average difference in viral abundance amounted to 1 × 10^5^ ± 8.1 × 10^4^ viruses mL^−1^ and 6.7 × 10^4^ ± 8.1 × 10^4^ viruses mL^−1^ in the Kermadec Trench and Atacama Trench, respectively, correspond to 28% and 9% lower viral abundance in samples fixed in situ vs. onboard from the two trench systems. As for the prokaryote counts, these differences in viral densities were not statistically significant (*p* > 0.05),

Four additional experiments assessing potential recovery artifacts were conducted using a pressure‐retaining chamber. The instrument was deployed to depths between 5400 m and 7770 m and retained around 65% of the in situ pressure after recovery. The difference between samples fixed in situ vs. onboard was in this case also statistically not significant (*p* > 0.05; paired two‐tailed Welch's *t*‐test), with an average value of 1.2 × 10^4^ ± 2.5 × 10^3^ more prokaryotes (72%) per mL^−1^ in samples fixed in situ compared to onboard.

The combined data set thus indicates that sample recovery from hadal depths has no statistically discernable effect on the quantification of either prokaryote or virus abundances, that is, any potential sampling bias would have been smaller than the variation in replicate measurements within a given sample.

### Abundance of prokaryotes and viruses in the water column

**Fig. 2:**
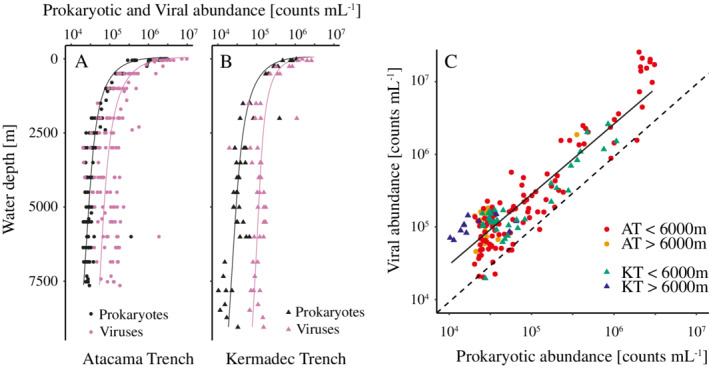
Water depth distributions of pelagic prokaryotic (black) and viral (pink) abundances in the Atacama Trench (**A**; circles; *n* = 131) and Kermadec Trench (**B**; triangles; *n* = 47). The regression equation of Atacama Trench prokaryotic (log10) and viral (log10) abundance as a function of oceanic depth (log10) was *y* = −0.67*x* + 5.6 (*R* ^2^ = 0.91) and *y* = −0.72*x* + 6.1 (*R* ^2^ = 0.80), respectively. In the Kermadec Trench, these fitted regressions were *y* = −0.66*x* + 5.6 (*R* ^2^ = 0.84) for prokaryotic abundance and *y* = −0.48*x* + 5.8 (*R* ^2^ = 0.66) for viral abundance. (**C**) Relationship between pelagic viral (*y*‐axis) and prokaryotic (*x*‐axis) abundances of the Kermadec Trench (green and blue triangles) and Atacama Trench (red and orange circles) with a fitted regression (black line) on log‐transformed data (*y* = 0.9871*x* + 0.5279; *R* ^2^ = 0.82; Pearson *P* < 0.001). The dotted line represents a 1 : 1 relationship.

The prokaryotic abundance in the water column exhibited similar decreases from the surface to abyssal depths across the nine hadal sites from the two trench systems, following a power function with numbers reaching from ~ 8.3 × 10^5^ prokaryotes mL^−1^ at 50 m to ~ 3.0 × 10^4^ prokaryotes mL^−1^ at ~ 4000 m (Fig. 2A,B). There were also no obvious difference in abundances between the five off‐axis sites and the trench sites to 6000 m depth (Fig. 2A,B; Supporting Information Fig. S2). In the Atacama Trench, prokaryotic abundance remained fairly stable from below 4000 m down to the trench bottom. However, in the Kermadec Trench, deeper values showed higher depth variation and a distinct fourfold decrease from 5.2 × 10^4^ ± 8.1 × 10^3^ prokaryotes mL^−1^ to 1.3 × 10^4^ ± 1.5 × 10^3^ prokaryotes mL^−1^ between 6000 and 7000 m (Fig. 2B).

Viral depth distributions were generally similar to those observed for prokaryotes (Fig. 2A,B), and accordingly, viral and prokaryotic abundances correlated in both trenches (Fig. 2C). In the Atacama Trench, the viral abundance at the surface was 2.4 × 10^6^ ± 3.3 × 10^5^ viruses mL^−1^ and it declined following a power function (*see* Fig. 2) to 8.3 × 10^4^ ± 5.8 × 10^3^ viruses mL^−1^ at 4000 m depth with almost no change in the average VP ratio (2.7 ± 0.2). However, at greater depths viral abundances and VP ratios gradually increased to 1.2 × 10^5^ ± 7 × 10^3^ viruses mL^−1^ and 4.3, respectively (see also Supporting Information Fig. S3). In the Kermadec Trench, the surface water contained about 2 × 10^6^ ± 4.2 × 10^5^ viruses mL^−1^ and the average VP ratio was 2.3 ± 0.4. As in the Atacama Trench, viral abundances in the Kermadec Trench declined with water depth, reaching 1.2 × 10^5^ ± 1.1 × 10^4^ viruses mL^−1^ at 4000 m, where the average VP ratio increased to 3.6 ± 0.3. Viral abundances in the Kermadec Trench remained fairly constant from 4000 m to the hadal ocean floor but, due to the distinct drop in prokaryotic abundances, the average VP ratios for hadal depths in Kermadec Trench were significantly higher (*p* > 0.05; two‐tailed Welch's *t*‐test) than in the Atacama Trench (6.5 ± 0.4 vs. 4.5 ± 0.4). In contrast, the average values for the VP ratios in the shallower realms did not differ significantly between the two trenches (epipelagic [0–1000 m] 1.8 ± 0.2 vs. 2.3 ± 0.3; bathyal [1000–4000 m] 2.4 ± 0.3 vs. 2.8 ± 0.3; and abyssal [4000–6000 m] 4.2 ± 0.4 vs. 3.8 ± 0.3; two‐tailed Welch's *t*‐tests, *p* < 0.05).

### Abundance of prokaryotes and viruses in sediments

#### Comparison of virus counts obtained by flow cytometry and epi‐fluorescence microscopy

**Fig. 3:**
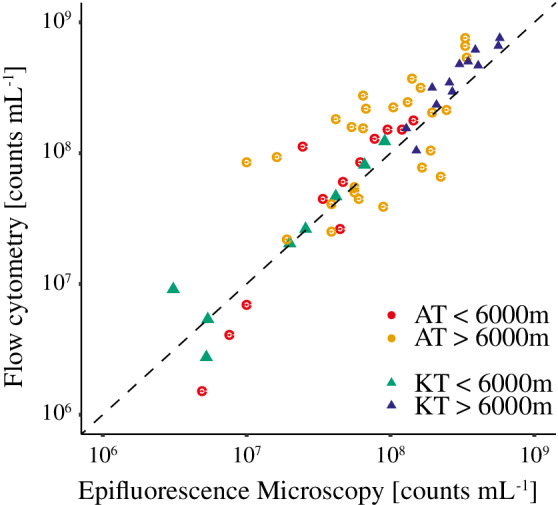
Comparison of benthic viral abundances quantified with epifluorescence microscopy and flow cytometry in sediments from the abyssal plain (red and green) and hadal sites (orange and blue) of the Atacama Trench (circles; *n* = 41) and Kermadec Trench (triangles; *n* = 20). The stippled line represents a 1 : 1 relationship. The equation of the linear regression between flow cytometry and epifluorescence microscopy counts (non‐log transformed) was *y* = 1.39*x* + 3.3*10^7^ (*R* ^2^ = 0.54, *p* ~ 2 × 10^−11^).

Flow cytometric approaches are routinely used to quantify viral abundances in pelagic samples, while epifluorescence microscopy has historically been favored for counting viruses in sediment samples (Suttle and Fuhrman 2010). To enable high sample throughput, we explored procedures for quantifying benthic viruses via flow cytometry. The developed approach was compared to standard epifluorescence microscopy counting for 61 individual samples extracted from sediment depths of 0–35 cm that were taken from a total of five different sediment cores recovered from the two trench systems. The values aligned reasonably well across three‐orders of magnitude showing a linear correlation with a low offset of around 39% ± 17% (*R* ^2^ = 0.54; Fig. 3). Thus, the offset indicated a small overestimation by flow cytometry as compared to natural sample‐to‐sample variation. Transmission electron microscopy imaging of selected samples resolved a large morphological diversity of viruses that included myoviridae, siphoviridae, and filamentous morphotypes (Supporting Information Fig. S4).

**Fig. 4:**
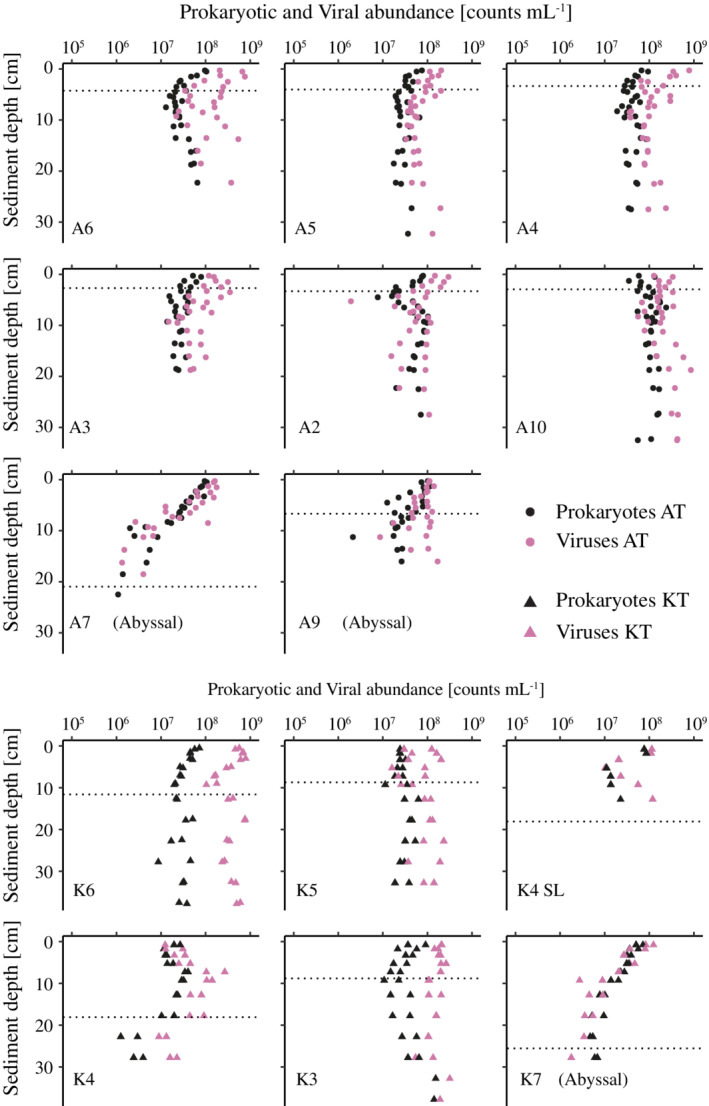
Sediment depth distributions of benthic prokaryotic (black) and viral abundances (pink) in cores from the Atacama Trench (circles; upper eight panels; *n* = 242) and Kermadec trench (triangles; lower six panels; *n* = 115). At the Kermadec Trench, only samples from K6, K4 SL, and K7 were without visual disturbances of the sediment surfaces. The dotted lines represent the oxygen penetration depths at the respective sites (Glud et al. 2021).

The prokaryotic abundances in the surface sediments (0–1 cm) along the trench axes of the Kermadec Trench and the Atacama Trench were similar, 5.7 × 10^7^ to 7.7 × 10^7^ prokaryotes mL^−1^ (*n* = 3) and 5.2 × 10^7^ to 1.3 × 10^8^ prokaryotes mL^−1^ (*n* = 12), respectively (Fig. 4). For the Kermadec Trench, we restricted this abundance assessment to sediment cores with intact surfaces (K4, K6). These two sites represented the higher and lower bounds of the observed range in in situ benthic O_2_ uptake along the trench axis (Glud et al. 2021) and we thus assume that they represent the full range of prokaryotic and viral abundance at our Kermadec Trench sites. Viral abundances were in a similar range of 1.1 × 10^8^ to 5.7 × 10^8^ viruses mL^−1^ and 1.1 × 10^7^ to 7.9 × 10^8^ viruses mL^−1^ for Kermadec Trench and Atacama Trench surface sediment, respectively (Fig. 4). Accordingly, the VP ratios ranged similarly in the sediment surfaces of the two trenches, albeit with relatively large within‐trench variability, 1.5–8.2 and 1.8–11.7 with average values of 5.8 ± 2.2 and 3.6 ± 0.8 in the Kermadec Trench and Atacama Trench, respectively.

The abyssal reference sites on the subducting plates (K7/A7) showed surface abundances of prokaryotes similar to those of their hadal counterparts (K7: 6.1 × 10^7^ prokaryotes mL^−1^, A7: 1 × 10^8^ prokaryotes mL^−1^), while abyssal viral abundances were lower (K7: 1 × 10^8^ viruses mL^−1^, Atacama Trench: 1.6 × 10^8^ viruses mL^−1^; Fig. 4). This pattern resulted in a lower VP ratio of 1.7 at K7 and 1.6 at A7.

**Fig. 5:**
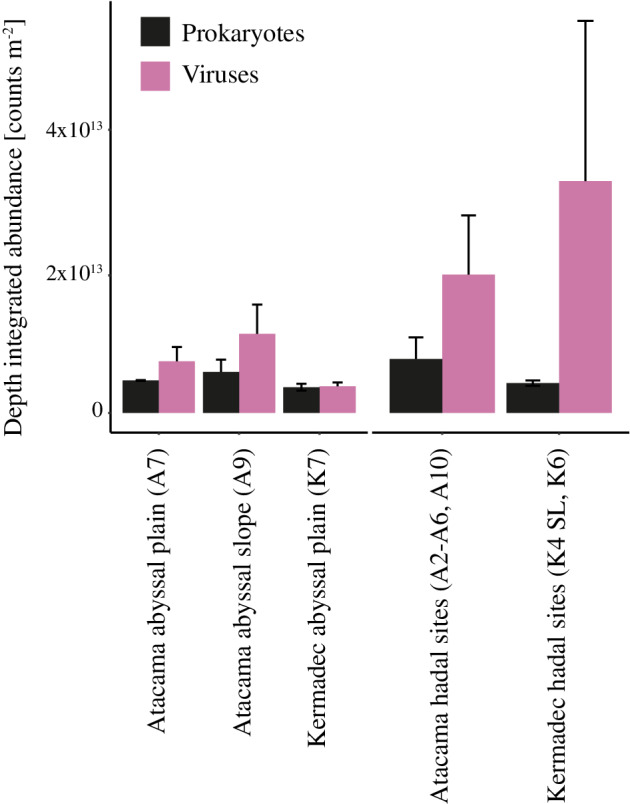
Mean depth integrated prokaryotic (black) and viral abundances (pink) to 15 cm depth at abyssal (left; each bar: *n* = 2) and hadal sites (right; Atacama Trench: *n* = 12; Kermadec Trench: *n* = 3) of the Atacama and Kermadec Trenches. Error bars indicate the standard error between individual sediment cores.

While surface abundances (0–1 cm) of prokaryotes and viruses were generally similar, vertical profiles of abundances differed markedly between abyssal and hadal sites. Abyssal sites had a quasi‐exponential decrease in abundances with sediment depth, reaching similar levels of 2.9 × 10^6^ ± 1.3 × 10^6^ prokaryotes mL^−1^ and 1.3 × 10^6^ ± 1.4 × 10^6^ viruses mL^−1^ at around 20 cm sediment depth at both K7 and A7, while the abundances in hadal sediments in the deeper layers fluctuated with depth and remained relatively high to the bottom of the cores (Fig. 4). There was generally a good agreement between replicate cores from given sites, but excursions or depth variation appeared site‐specific (Fig. 4). At the abyssal landward site of the Atacama Trench (A9), prokaryotic and viral abundances also decreased with sediment depth, but values appeared more disturbed than at the subducting plate sites (A7/K7) (Fig. 4). Depth‐integrated prokaryotic abundances in the upper 15 cm (the maximum depth in the shortest cores) were around 19% and 66% higher in the hadal sediment of the Kermadec and Atacama Trenches, respectively, than in the adjacent abyssal sediment (Fig. 5). Furthermore, more prokaryotes were present in the upper 15 cm of hadal sediments in the Atacama Trench (7.8 × 10^12^ ± 1.2 × 10^12^ prokaryotes m^−2^) than in the Kermadec Trench (4.5 × 10^12^ ± 2.6 × 10^11^ prokaryotes m^−2^; *p* < 0.05, two‐tailed Welch's *t*‐test). In contrast, integrated viral abundances did not differ significantly between the Atacama Trench and Kermadec Trench (2 × 10^13^ ± 3.7 × 10^12^ viruses m^−2^ vs. 4.1 × 10^13^ ± 1.6 × 10^13^ viruses m^−2^), respectively; *p* > 0.05, two‐tailed Welch's *t*‐test.

**Fig. 6:**
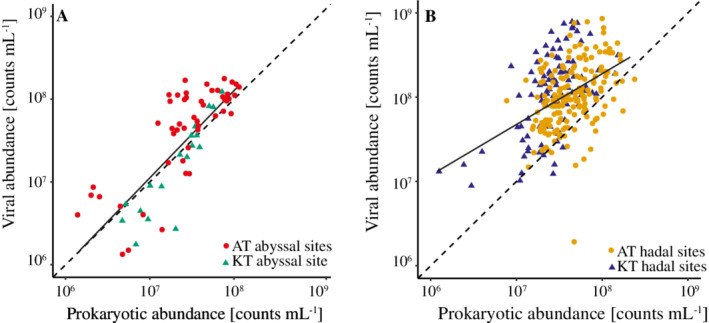
Comparison of benthic viral and prokaryotic abundances in sediment at abyssal sites (**A**) (*n* = 76) and hadal sites (**B**) (*n* = 281) of the Kermadec Trench (triangles) and the Atacama Trench (circles). The stippled lines represent a 1 : 1 relationship. The equation for the regression between log‐transformed viral and prokaryotic abundance was *y* = 1.05*x* − 0.27 (*R* ^2^ = 0.64, Pearson *P* < 0.001) in abyssal settings and *y* = 0.62*x* + 3.35 (*R* ^2^ = 0.20, Pearson *P* < 0.001) for hadal samples.

While prokaryotic and viral abundances showed a strong positive correlation at the abyssal sites (Fig. 6A), such a relation was less clear for the hadal sediment (Fig. 6B). The viral and prokaryotic abundances showed strong correlations in 10 out of 21 individual hadal sediment cores (*R* ^2^ ≥ 0.4, Supporting Information Fig. S5), but the relation was only weakly correlated across the entire hadal data set (Fig. 6B; *R* ^2^ = 0.20, Pearson *P* < 0.001). These findings indicated a close coupling between viruses and prokaryotes at some sites, yet a large overall variation in VP ratios in the sediment from both hadal regions.

**Fig. 7:**
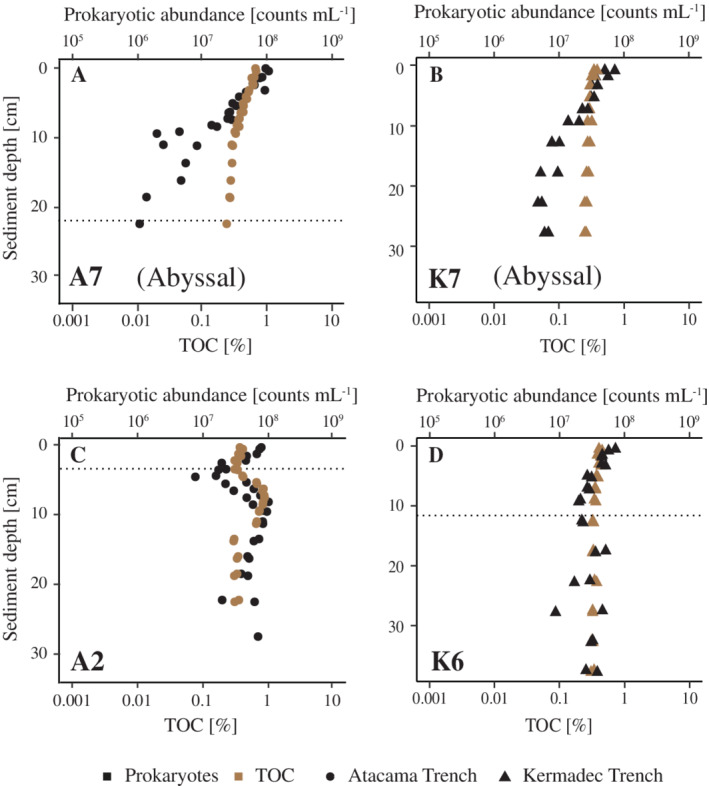
Sediment depth distributions of prokaryotic abundance (black) and TOC (brown; % dry weight) profiles from selected sites from the Kermadec Trench (triangles) and Atacama Trench (circles) (**A**–**D**).

**Fig. 8:**
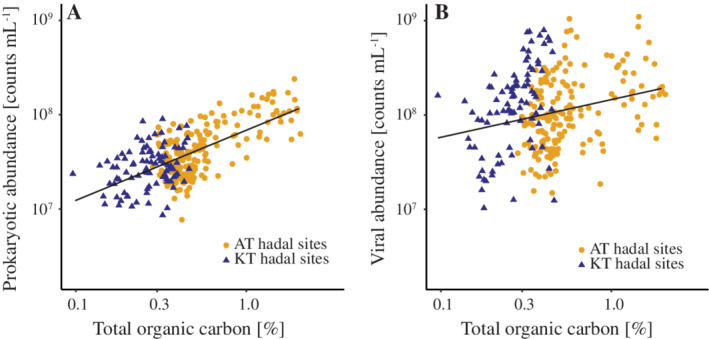
Relationships of prokaryotic (**A**) and viral (**B**) abundances (*y*‐axes) and the total organic content (TOC) (*x*‐axes) in sediments from the Atacama Trench (orange circles; *n* = 174) and Kermadec Trench (blue triangles; *n* = 88). The equation for the linear regressions between log‐transformed prokaryotic abundances and TOC was *y* = 0.75*x* + 7.86 (*R* ^2^ = 0.43) and between log‐transformed viral abundances and TOC concentrations *y* = 0.39*x* + 8.17 (*R* ^2^ = 0.05).

The benthic TOC profiles also differed markedly between abyssal and hadal settings. In abyssal sediments, the TOC content decreased quasi‐exponentially (Fig. 7A,B) while the hadal site exhibited highly variable levels with occasional subsurface peaks (Fig. 7C,D; Supporting Information Fig. S6). These subsurface peaks of TOC were similar to those of prokaryotic abundance and the two parameters correlated well (*R* ^2^ = 0.54; Pearson *P* < 0.001) over the entire range of around ~ 0.1–2% TOC availability that was measured in hadal samples of both the Kermadec and Atacama Trenches (Fig. 8A). In contrast to this result, viral abundances were more variable and showed no clear relation to the TOC availability (*R* ^2^ = 0.04; Pearson *P* > 0.001) in the hadal samples of this study (Fig. 8B).

## Discussion

### Determination of prokaryotic and viral abundances in hadal systems

The in situ fixation experiments allowed the potential effects of water sample recovery from hadal depths on prokaryotic and viral abundances to be assessed for the first time. Data from these experiments demonstrated that decompression and transient heating during sample recovery had no statistically significant effect on prokaryotic and viral abundances. In situ fixation experiments were solely conducted on pelagic samples. However, we speculate that these results also apply for benthic environments, even though benthic and pelagic hadal environments are characterized by different prokaroytic communities (Peoples et al. 2019*b*). Based upon the decompression of a piezophilic pure culture, Chastain and Yayanos (1991) suggested that samples from the hadal zone need to be either fixed in situ or rapidly after recovery. From an environmental perspective, it is well documented that benthic samples recovered from great depths exhibit shifts in metabolic activity (Glud et al. 1994; Tamburini et al. 2013) and solute concentrations (Hall et al. 2007), which are presumably associated with leakage of intracellular material (Glud et al. 1994; Hall et al. 2007). However, the observed lack of recovery artifacts on prokaryotic cell abundance indicated that even without in situ fixation, stainable nucleic acids remain enveloped and can be reliably counted by epifluorescence microscopy or flow cytometry, despite disrupted or changed metabolic performance.

For this study, we applied flow cytometry for quantifying prokaryotic and viral abundances in extracts derived from sediment samples using a new approach that enabled high sample throughput and yielded an unprecedented amount of data from hadal sediments. Evaluation of viral extracts using transmission electron microscopy confirmed a high purity of viruses in comparison to sediment particles, and demonstrated the presence of diverse tailed and filamentous morphotypes as previously observed in other marine sediments (Middelboe et al. 2003).

The filamentous morphotypes were not detected by epifluorescence microscopy and thus they are likely also not captured by flow cytometry. Consequently, our quantification of viral abundance in sediments might be underestimated if these filaments are indeed viruses. It has been speculated that for environments with little potential for viral spreading, such as sediments, the chronic infections, that are typical of filamentous single‐stranded DNA viruses (Rakonjac et al. 2011), might be an advantageous strategy for viral replication compared to lytic infections that kill the viral host (Middelboe et al. 2001; Martiny et al. 2014). In a metagenomic study on viral diversity in hadal sediments, only 24–30% of sequences showed similarities to known viral proteins in public databases (Yoshida et al. 2013). Hence, the majority of viral diversity—as well as the relative importance of different viral morphotypes and infection strategies in marine sediments—remains to be investigated.

### Prokaryotic and viral abundances in the water column

The profiles of pelagic prokaryotic abundance in the upper ~ 3000 m of the water column resembled the simple power functions that are generally used to describe particulate organic carbon fluxes (Martin et al. 1987). Indeed, the exponents for both prokaryotic (Atacama Trench: 0.67, Kermadec Trench: 0.66; *see* Fig. 2) and viral abundances (Atacama Trench: 0.72, Kermadec Trench: 0.48) were in the range of what has been derived for the vertical carbon flux in the two areas (~ 0.7) (Henson et al. 2012), suggesting that prokaryotic and viral abundances in the upper layers are linked to the vertical flux of particulate organic carbon. The low variability in abundances in deeper waters presumably reflected the constant low concentration of bioavailable organic carbon, which is also evident in the invariable dissolved organic carbon concentrations that are generally observed in the mesopelagic and abyssal realms (Bendtsen et al. 2002; Lønborg et al. 2018). While most previous studies only reached depths of ~ 4000 m, we sampled the entire water column and showed that prokaryotic and viral abundances remained at a relatively stable level down to hadal depths in the Atacama Trench region. However, in Kermadec Trench waters, prokaryotic abundance dropped abruptly at around 6000 m, which coincided with the rim of the trench. This observation was consistent across three separate sites. Similarly, a large variability of prokaryotic abundances was found in the Kermadec Trench by Peoples et al. (2018), with numbers ranging around 1.4 × 10^4^ to 3.3 × 10^4^ prokaryotes mL^−1^ and a notable shift in community composition was observed in samples below 6000 m (Peoples et al. 2018). These patterns might imply a different water mass within the trench with lower substrate availability, but at present we have no hydrographic data nor DOC measurements from the hadal zone in the Kermadec Trench to explore this contention any further.

The observed prokaryotic abundances at hadal depths (Atacama Trench: ~ 4.1 × 10^4^ prokaryotes mL^−1^; Kermadec Trench: ~ 2.5 × 10^4^ prokaryotes mL^−1^) were similar to values reported in previous studies by Peoples et al. (2018) (Kermadec Trench: ~ 2.1 × 10^4^ prokaryotes mL^−1^, Mariana Trench: ~ 1.3 × 10^4^ prokaryotes mL^−1^), yet higher than those previously obtained from the Japan Trench (~ 1.1 × 10^4^ prokaryotes mL^−1^) (Nunoura et al. 2016) and the Mariana Trench (~ 6.4 × 10^3^ prokaryotes mL^−1^) (Nunoura et al. 2015). We cannot exclude that these differences arose from the fact that different procedures were applied in the respective studies. However, the difference in procedures should not have affected the relative differences between the Atacama Trench and Kermadec Trench and Mariana Trench (this study and Peoples et al. 2018), as well as between the Mariana and Japan trenches (Nunoura et al. 2015, 2016). The primary production in the overlying water column of the Japan Trench (~ 760 mg C m^−2^ d^−1^) is intermediate between estimates for the Kermadec Trench (380 mg C m^−2^ d^−1^) and Atacama Trench (910 mg C m^−2^ d^−1^), while the Mariana Trench is overlain by very oligotrophic waters with lower rates (~ 120 mg C m^−2^ d^−1^). All net primary production values were derived as described in Lutz et al. (2007) by applying remote sensor data collected from 2009 to 2018. Thus, prokaryotic abundance in the underlying hadal realm appears to be linked to surface production. One potentially important transport mechanism that is known to rapidly connect primary production in the euphotic zone with deeper reaches of the ocean is sinking aggregates (Shanks and Trent 1980; Iversen and Ploug 2010), which presumably is an important source of labile organic material that sustains prokaryotic abundances (Turley and Mackie 1994). We therefore argue that more investigations using standardized quantification procedures and sophisticated sampling techniques that include sinking particulate organic carbon in different trench settings are needed to explore linkages between nutrition levels at the ocean surface, particle transport, substrate availability, and pelagic prokaryotic abundance in the hadal realm.

Viral abundances generally followed the same depth distribution as prokaryotic abundances but exhibited higher variation. The resulting VP ratios thus ranged 30‐fold from 0.3 to 12.6 and increased with water depth (*see* Supporting Information Fig. S6). A depth increase in VP ratios has previously been ascribed to a gradual decrease in viral decay associated with lower ambient temperatures (Parada et al. 2007; Suttle 2007). However, water temperature decreases only until ~ 4500 m depth and rises thereafter approximately 0.16°C every 1000 m (Blankenship et al. 2006; Jamieson et al. 2011). Hence, our data might imply increased virus‐driven lysis of prokaryotes with increasing water depth. This explanation aligns with previous studies arguing that low prokaryotic abundances make eukaryotic grazing energetically unfavorable, thus reducing energy transport to higher trophic levels and leaving viruses as the dominant mortality factor for prokaryotes in low cell density environments (Patterson et al. 1993; Magagnini et al. 2007; Pachiadaki et al. 2016).

**Fig. 9:**
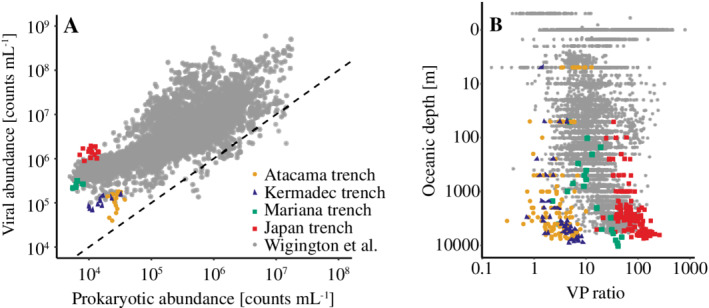
(**A**) Relationship between pelagic viral and prokaryotic abundances from hadal depths from the Mariana Trench (green squares, *n* = 6; Nunoura et al. 2015), Japan Trench (red squares, *n* = 16; Nunoura et al. 2016), Kermadec Trench (blue triangles, *n* = 13; this study), and Atacama Trench (orange circles, *n* = 24; this study), in comparison with a global compilation of abundance data (gray circles, Wigington et al. 2016). The stippled line represents a 1 : 1 relationship. (**B**) VP ratios with oceanic depth of all datapoints from the aforementioned studies.

Based on a global compilation of more than 5500 pelagic datapoints, Wigington et al. (2016) concluded that VP ratios increase with decreasing prokaryotic abundances corresponding to an incline from ~ 24 at the surface ocean to ~ 46 at abyssal water depths. However, 80% of the compiled data originated from water depths above 500 m, and the assessments only reached a maximum depth of 6000 m with the relatively few deep‐sea values showing little variation (Fig. 9). The hadal VP ratios of our study ranged from 1.7 to 8.6 with a relatively low average value of 5.2. However, that data align with values of Hara et al. (1996), in which VP ratios ranged from 1.0 to 8.7 for water depths from 500 to 5000 m and with values of Weinbauer et al. (2003), who observed ratios around 1.1–5.6 in the depth range of 800–2000 m. Both of these data sets were not included in Wigington et al. (2016). To our knowledge, the only other available data sets from hadal depth show VP ratios of 4.5–48.5 (average 34.1) in the Mariana Trench (Nunoura et al. 2015) and 74.3–258.1 (average 140.8) in the Japan Trench (Nunoura et al. 2016). Even though we cannot exclude that the different ranges might partly relate to the fact that the respective studies applied different measuring procedures, the available hadal data suggest a continued gradual increase in VP ratios from the bathypelagic into the hadal realm (Fig. 9). The relatively high variation in hadal VP ratios resembles the variations encountered in the surface ocean (Wigington et al. 2016), implying that conditions in hadal settings are also highly variable.

### Hadal sediments show high abundances and spatial heterogeneity of prokaryotes and viruses

The current study supports previous observations of elevated abundances of prokaryotes (Glud et al. 2013; Manea et al. 2019; Hiraoka et al. 2020) and viruses (Danovaro et al. 2016; Manea et al. 2019) in hadal sediments as compared to adjacent abyssal settings. These observations were hypothesized to mirror an enhanced flux of organic matter to the hadal benthos, stimulating prokaryotic activity (Glud et al. 2013; Wenzhöfer et al. 2016), and a potential important regulating role of viruses (Manea et al. 2019). However, these previous studies were restricted to single sampling sites in the central sedimentation basins of a few trenches (e.g., Izu‐Bonin Trench, Wenzhöfer et al. 2016; Manea et al. 2019; Tonga Trench, Wenzhöfer et al. 2016; and the Challenger Deep of the Mariana Trench, Glud et al. 2013). Here, we targeted a series of sites on 430 km and 210 km long transects along the Atacama Trench and Kermadec Trench axes, respectively, to resolve the spatial variation of prokaryotic and viral abundance within and between two trench systems and to explore potential relations to substrate availability. There was no apparent large‐scale variation along the two transects within the two trenches. Yet notably, we observed extensive and erratic downcore variations in prokaryotic abundances in all hadal sites as compared to the steady exponential decline observed at the abyssal reference sites. The latter pattern presumably reflected the gradual downcore depletion in labile organic carbon typically observed in abyssal sediments (Witbaard et al. 2000). The prokaryotic abundance in the hadal sediments also covaried with the TOC availability and downcore variation in abundance was thus also likely driven by substrate availability.

The downcore distribution of TOC in both hadal trenches likely reflected highly irregular deposition, presumably as a consequence of mass wasting events. Erratic downslope mass wasting of sediments and TOC might have resulted in the import of prokaryotes and viruses into the hadal benthos and could have left the site‐specific imprints we observed at each of the hadal sites. Alternatively, the dramatic perturbation could lead to enhanced substrate availability in the hadal realm as benthic surface material and infauna from shallower sediment are washed into the trench interior, boosting prokaryotic activity and viral infections in niches of enrichments (Oguri et al. 2013). Recent studies point to local population dynamics rather than import as the main cause of downcore fluctuations in prokaryotic and viral abundances. That is, investigations of prokaryotic community composition indicate that deep trench prokaryotic communities are distinct to their adjacent abyssal counterparts and change gradually over sediment depth (Peoples et al. 2019*b*; Hiraoka et al. 2020). This suggested that the prokaryotes transported and buried in mass wastings were probably inhibited or lysed, and likely boosted the activity of distinct hadal communities.

Bursts in benthic activity and prokaryotic growth are typically associated with enhanced virus production (Glud and Middelboe 2004). For instance, detailed investigations of microscale dynamics around patches of organic carbon enrichment in coastal sediments have shown how increased abundance in prokaryotes is followed by a delayed enhancement in virus production (Carreira et al. 2013). This dynamic led to an increase in the VP ratio that gradually reverted to the original value and thereby a transient uncoupling in the otherwise stable relation between prokaryotic and viral abundances (Carreira et al. 2013). Similar, seasonal deposition dynamics of phycodetrital material have been shown to drive extensive temporal variations in the benthic VP ratio (Siem‐Jørgensen et al. 2008). Thus dynamics in organic matter supply are expected to induce variations in the VP ratio and a poor overall correlation between the abundance of prokaryotes and viruses, while stable conditions maintain a relatively constant VP ratio. The time scales of such uncouplings will presumably depend on the degree of perturbation and the overall metabolic activity of the system.

These factors likely explain the strong correlation between the abundance of prokaryotes and viruses at the stable and undisturbed abyssal sites, while mass wastings and stochastic local organic matter enrichments may have led to a temporal and spatial uncoupling of the abundance of prokaryotes and viruses in hadal settings as compared to abyssal sites. This difference suggests that the relationship between viruses and their prokaryotic hosts in hadal sediments is much more dynamic than in abyssal sediments, and is probably driven by the spatiotemporal depositional dynamics in hadal trenches. The enriched substrate conditions in hadal sediments likely promote more virus‐induced prokaryotic mortality and recycling of organic material in dynamic hadal settings as compared to the stable and substrate limited conditions at abyssal plains. Ultimately our results suggested that conditions in central trench basins may poorly represent the hadal realm in general (Wenzhöfer et al. 2016; Stewart and Jamieson 2018). However, most of the variability within trenches is found in downcore patterns on small spatial scales—not on larger geographic scales along the trench axes.

## Conflict of Interest

None declared.

## Supporting information


**Appendix S1.** Supporting Information.
