# Supplementary material for: Spatial variability of prokaryotic and viral abundances in the Kermadec and Atacama Trench regions
Source: Limnol Oceanogr. 2021 Feb 28;66(6):2095–109. doi: 10.1002/lno.11711 (PMC8248377; doi:10.1002/lno.11711)
Supplement: Supplementary file 1 — Appendix S1. Supporting Information. [file LNO-66-2095-s001.pdf]

Supplementary Information

Spatial variability of prokaryotic and viral abundances in the Kermadec and Atacama trench regions

Supplement Figures and Tables:

Supplement Table 1. Settings for flow cytometry measurements.

Sample	FSC	SSC	SYBR	Flow rate
Water column	404	572	525	medium
Sediment (Prokaryotes)	473	593	555	low
Sediment (Viruses)	404	555	525	low

Supplement Table 2. Overview of *in situ* fixation data

	experiment #	fixative	trench	depth	*in situ* fixed (× 10^4 mL^{-1}) average abundance	*in situ* fixed (× 10^4 mL^{-1}) average SD	control (× 10^4 mL^{-1}) average abundance	control (× 10^4 mL^{-1}) average SD	differences (× 10^4 mL^{-1}) *in situ* vs control	differences (× 10^4 mL^{-1}) average
Syringe system prokaryotes	1	GLT	Kermadec	8721	8.28	1.39	7.44	0.44	0.84	
	2	GLT	Kermadec	6842	5.06	1.93	5.29	1.53	-0.24	
	3	GLT	Kermadec	5950	3.17	1.36	8.52	11.30	-5.35	
	4	GLT	Kermadec	8721	5.75	5.67	3.59	6.35	2.16	-1.14
	5	GLT	Kermadec	8721	2.52	0.26	2.42	0.47	0.10	
	6	GLT	Kermadec	7930	0.98	0.25	4.41	0.25	-3.43	
	7	GLT	Kermadec	8721	0.89	0.48	2.98	0.21	-2.10	
Syringe system viruses	1	GLT	Kermadec	8721	7.37	4.97	5.22	2.11	2.15	
	2	GLT	Kermadec	6842	16.87	7.00	5.31	7.43	11.56	
	3	GLT	Kermadec	5950	33.52	5.01	86.40	76.47	-52.88	
	4	GLT	Kermadec	8721	46.35	40.37	41.39	45.09	4.95	-10.64
	5	GLT	Kermadec	8721	30.13	8.85	43.54	14.43	-13.41	
	6	GLT	Kermadec	7930	34.00	2.63	42.54	3.24	-8.54	
	7	GLT	Kermadec	8721	23.32	3.01	41.65	4.77	-18.32	
Syringe system prokaryotes	8	PFA	Atacama	7767	2.63	0.28	3.07	0.40	-0.44	
	9	PFA	Atacama	7638	12.50	0.54	12.97	1.27	-0.47	
	10	PFA	Atacama	7638	4.87	0.13	5.42	0.49	-0.55	-2.44
	11	PFA	Atacama	6945	17.53	0.64	21.93	0.94	-4.40	
	12	PFA	Atacama	7638	15.42	0.46	21.76	1.17	-6.34	
Syringe system viruses	8	PFA	Atacama	7767	22.35	9.04	12.67	6.42	9.67	
	9	PFA	Atacama	7638	40.19	5.88	61.59	16.64	-21.40	
	10	PFA	Atacama	7638	60.92	15.13	62.23	19.02	-1.31	-6.70
	11	PFA	Atacama	6945	123.58	22.46	114.03	16.34	9.56	
	12	PFA	Atacama	7638	73.21	18.61	103.23	33.12	-30.02	
PRS prokaryotes	13	PFA	Atacama	6000	1.47		0.88		0.59	
	14	PFA	Atacama	5400	2.32		1.31		1.01	0.94
	15	PFA	Atacama	6945	3.35		1.35		2.00	
	16	PFA	Atacama	7850	1.89		1.72		0.17	

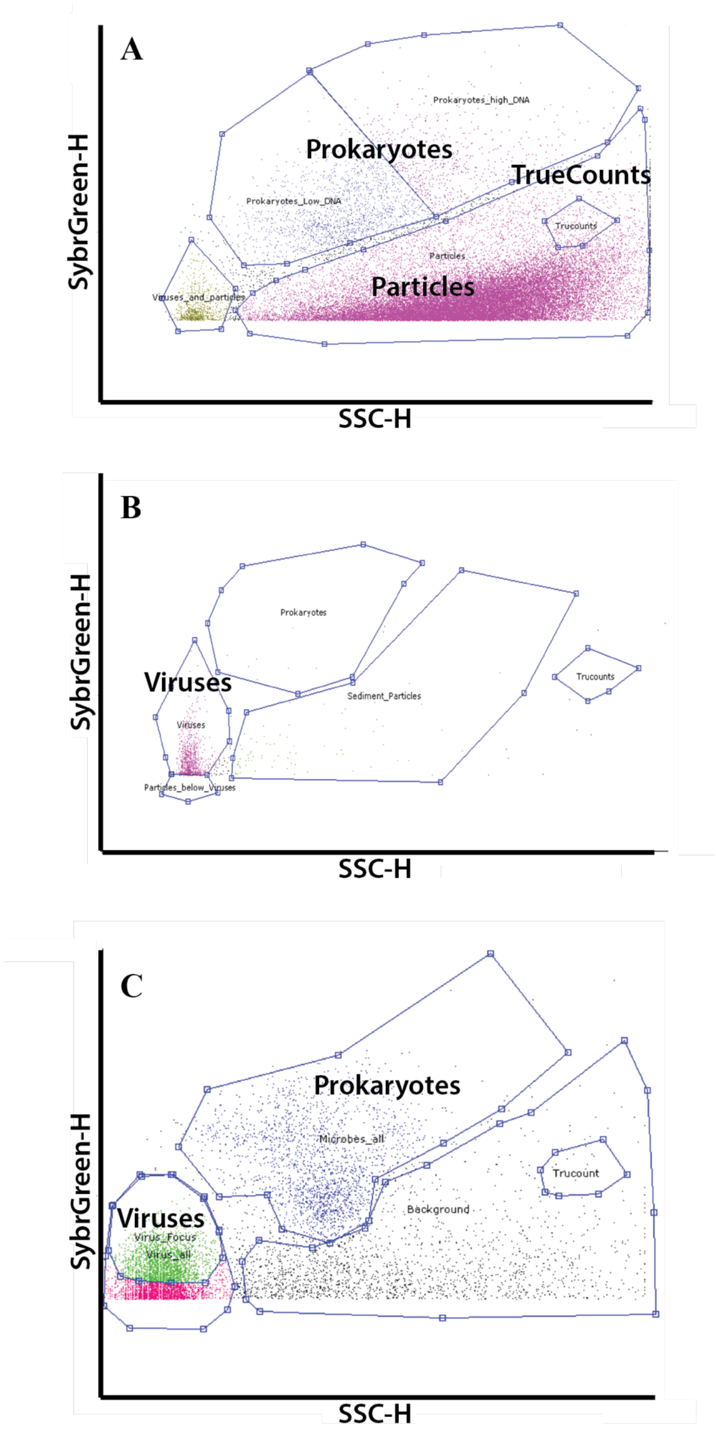

Supplement Figure 1. Gating examples for flow cytometry data of prokaryotic abundance in the sediment **(A)**, viral abundance in the sediment **(B)** and prokaryotic and viral abundance in the water column **(C)**, taken as screenshots from the Flowing Software (Terho, 2013).

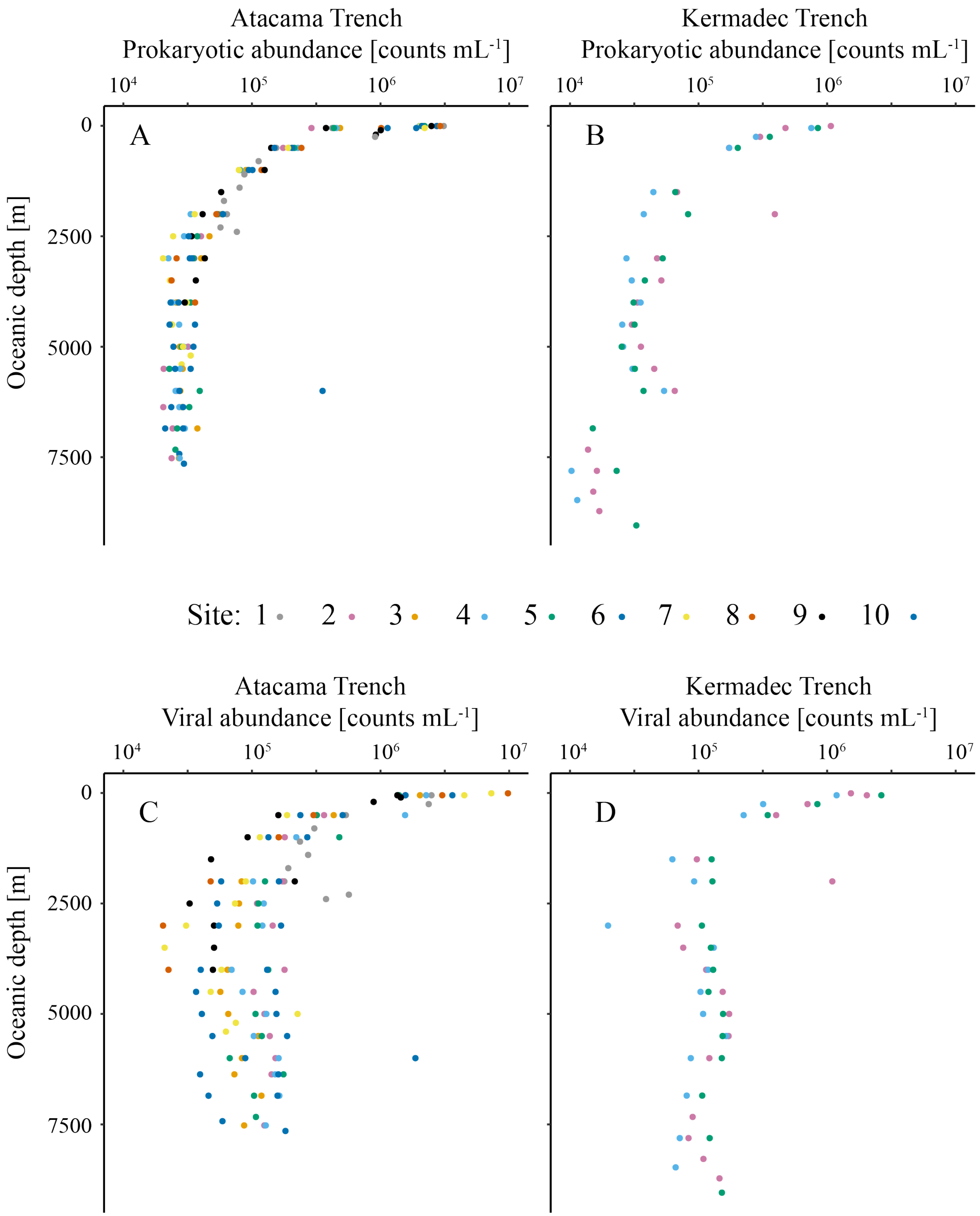

Supplement Figure 2. Pelagic prokaryotic **(A & B)** and viral abundances **(C & D)** with water depth (y axis) at each sampling site (colors) in the Atacama Trench **(A & C)** and Kermadec Trench **(B & D)**.

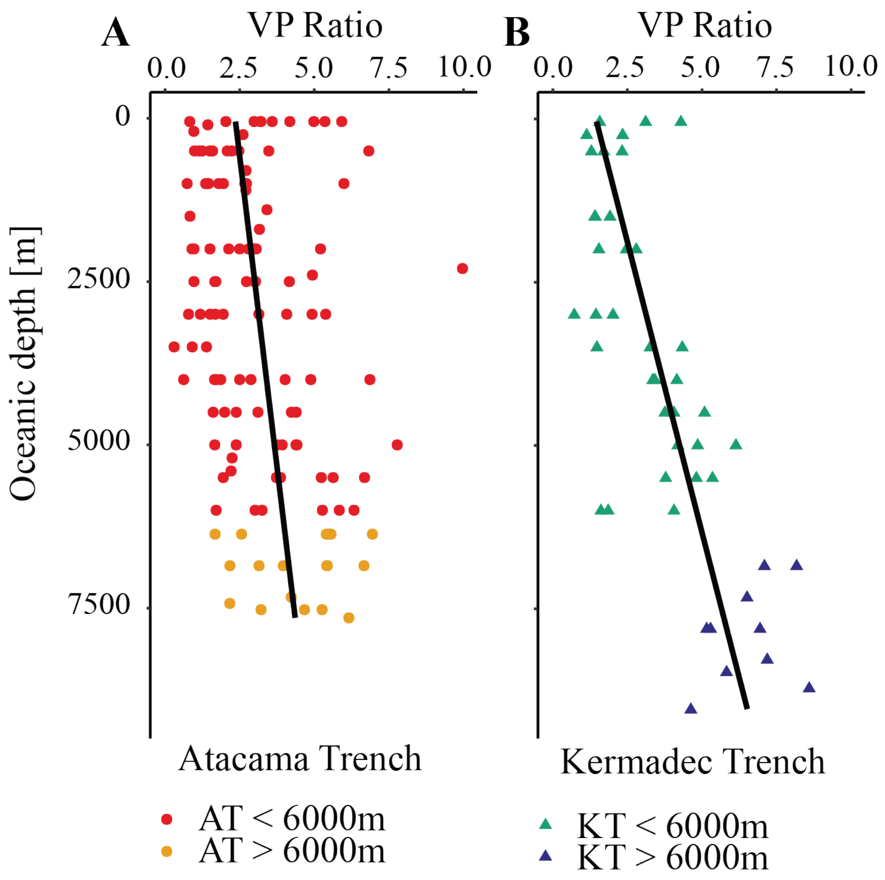

Supplement Figure 3. Pelagic VP ratios (x axis) from the Atacama Trench **(A)** and Kermadec Trench **(B)** with oceanic depth (y axis). The equations for linear correlations were $y = 2.6*10^{-4}x +2.3$ ($R^2 = 0.1$, $p \sim 0.0002$) in the Atacama Trench and $y= 5.6*10^{-4}x +1.4$ ($R^2 = 0.56$, $p \sim 1.3 \times 10^{-9}$) in the Kermadec Trench. The black lines represent the fitted linear regressions.

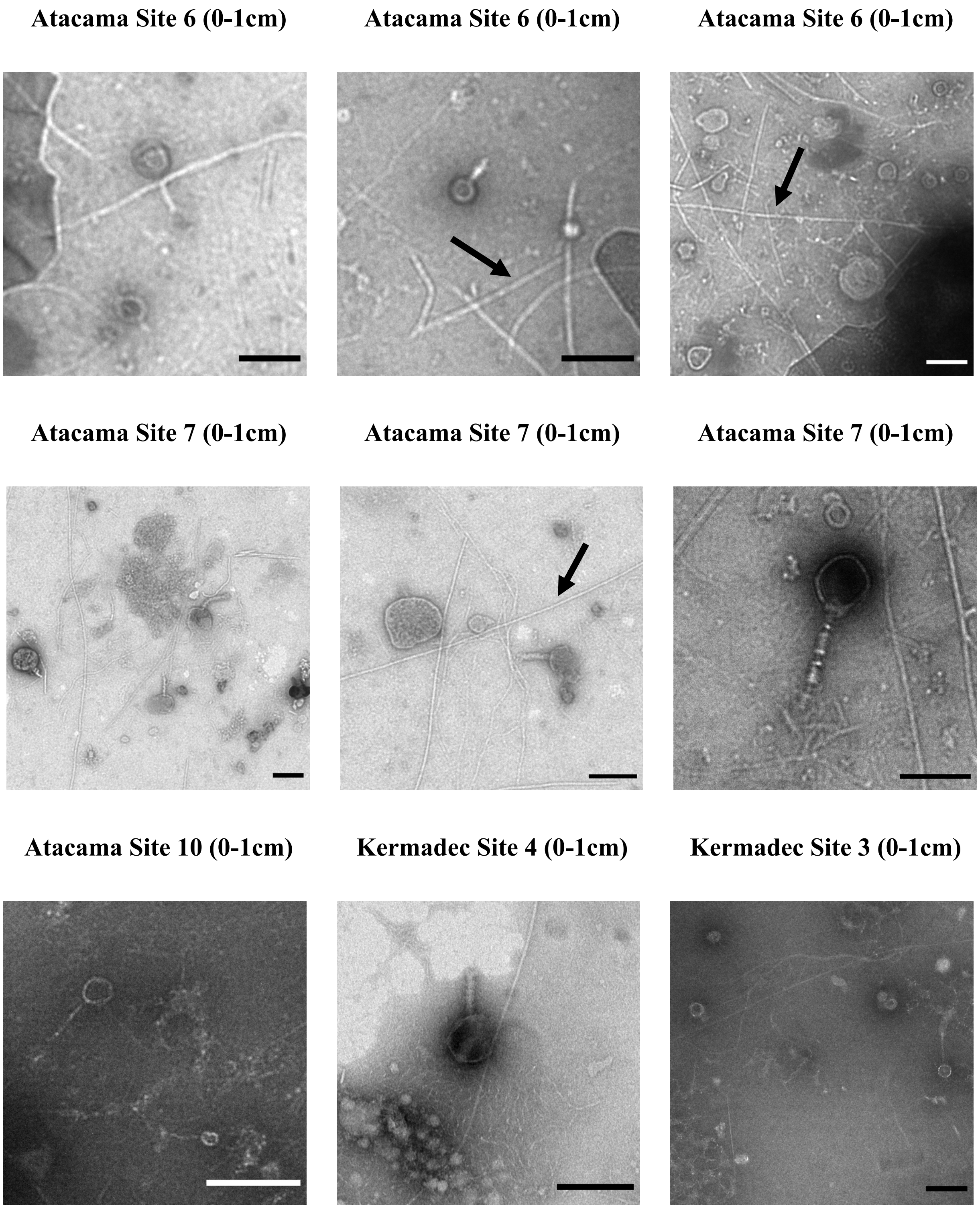

Supplement Figure 4. Examplary transmission electron microscopy pictures taken from virus extracts of hadal and abyssal sediments. All scale bars are 200 nm wide. Examples of potential filamentous morphotypes are indicated by the black arrows.

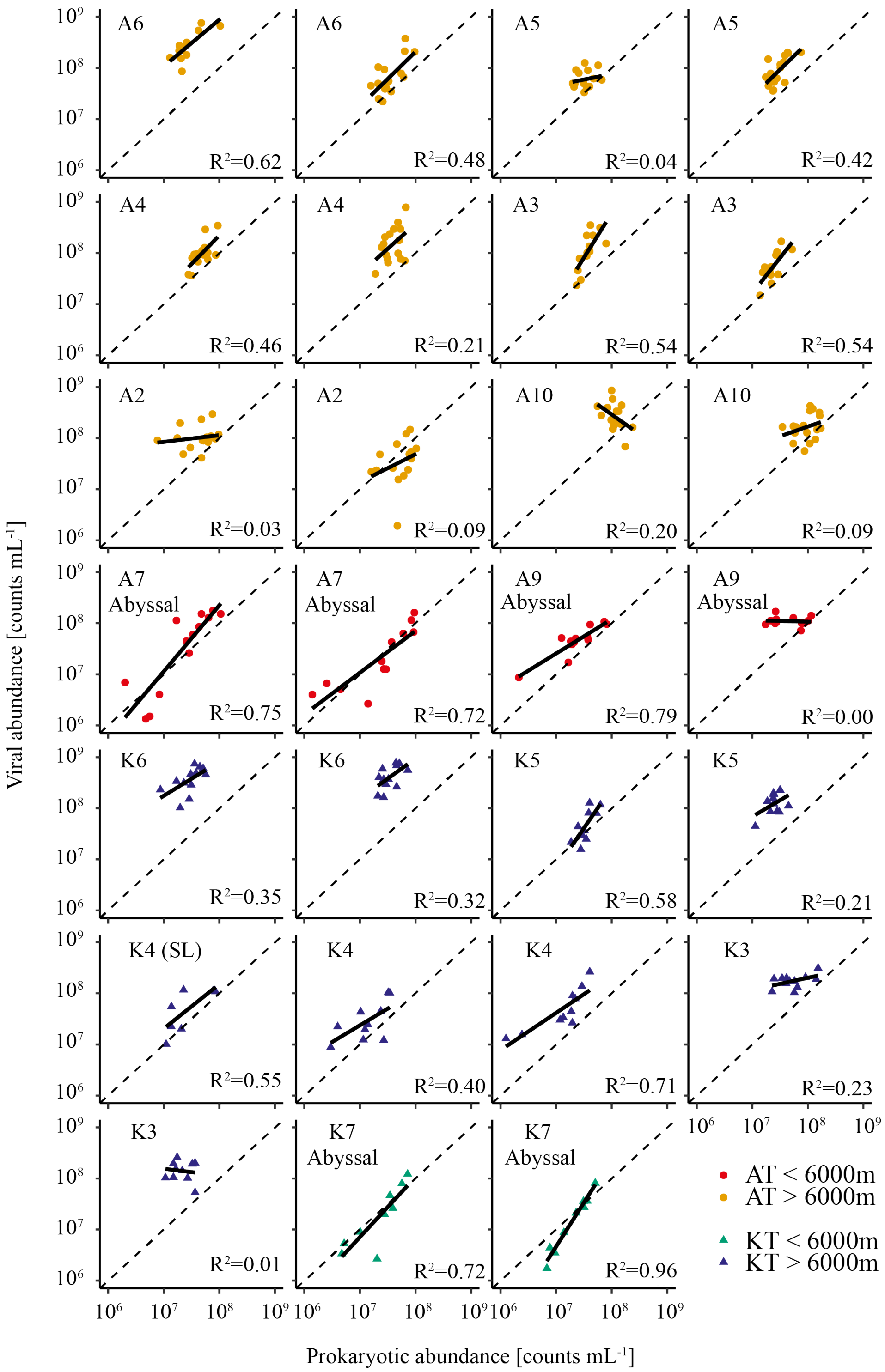

Supplement Figure 5. Benthic viral (y axes) and prokaryotic (x axes) abundances of individual sediment cores from each individual sampling site of the Atacama Trench (upper 16 panels) and Kermadec Trench (lower 11 panels). The R^2 values depict the variation in Pearson correlations of viral and prokaryotic abundances (log transformed data).

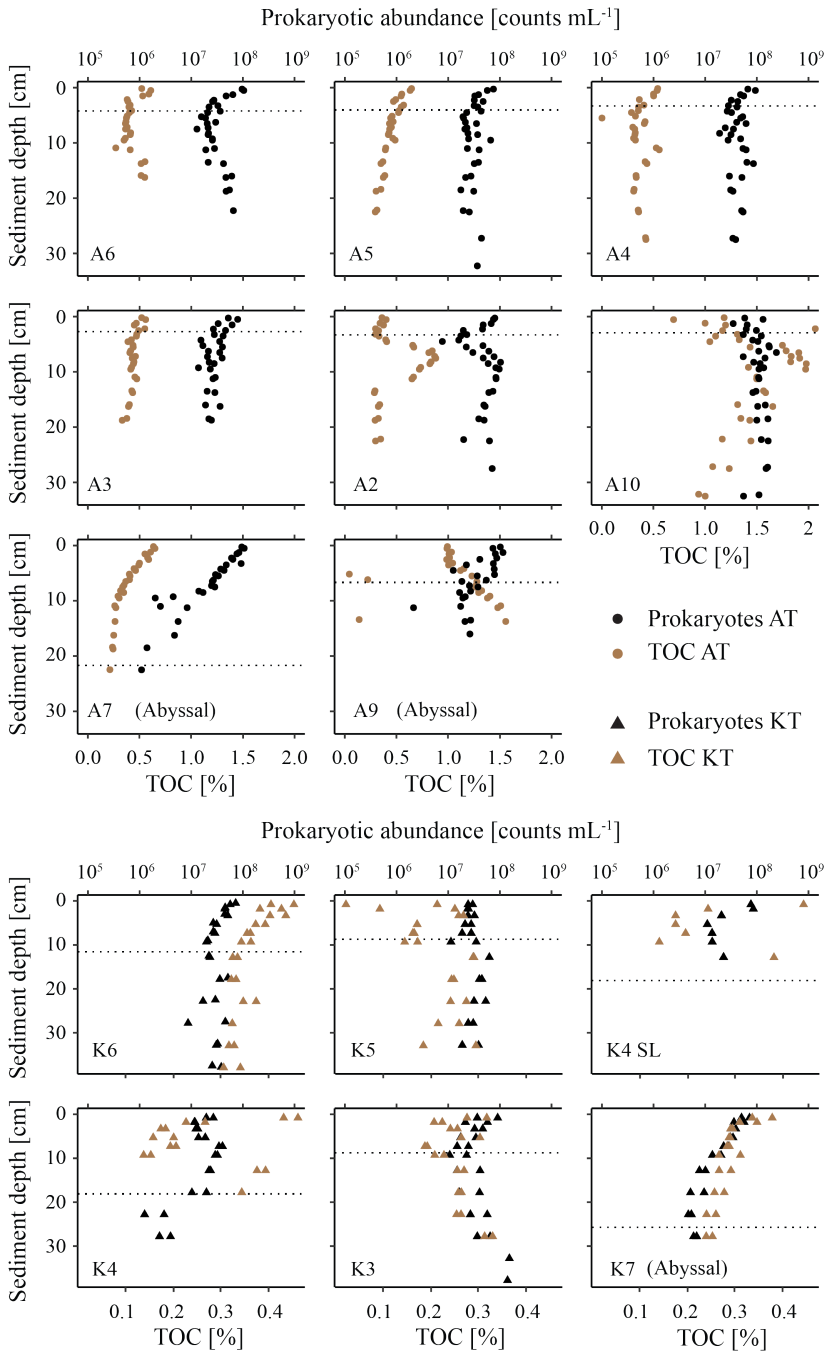

Supplement Figure 6. Benthic prokaryotic abundance (black) and Total Organic Carbon (TOC) concentrations (brown) in sediments of the Atacama Trench (upper eight panels) and Kermadec Trench (lower six panels). At the Kermadec Trench, only samples from K6, K4 SL and K7 were without visual disturbances of the sediment surfaces. The dotted lines represent the oxygen penetration depths at the respective sites (Glud *et al.*, in press).
